# Genotyping indicates marked heterogeneity of tuberculosis transmission in the United States, 2009–2018

**DOI:** 10.1017/S0950268821002041

**Published:** 2021-09-14

**Authors:** Carly A. Rodriguez, Tenglong Li, Julie L. Self, Helen E. Jenkins, Charles R. Horsburgh, Laura F. White

**Affiliations:** 1 Department of Epidemiology, Boston University School of Public Health, Boston, MA, USA; 2 Department of Biostatistics, Boston University School of Public Health, Boston, MA, USA; 3 Division of Tuberculosis Elimination, National Center for HIV/AIDS, Viral Hepatitis, STD, and TB Prevention, Centers for Disease Control and Prevention, Atlanta, GA, USA

**Keywords:** epidemiology, tuberculosis (TB)

## Abstract

Heterogeneity in the number of secondary tuberculosis (TB) cases per source case, the effective reproductive number, R, is important in modelling prevention strategies' impact on incidence.

We estimated mean *R* (*R*_m_) and calculate the dispersion parameter of this distribution, *k*, using surveillance and genotyping data for U.S. cases during 2009–2018. We modelled transmission assuming cases in a cluster have matching genotypes and share characteristics related to geography, temporal proximity (i.e. serial interval) and time since U.S. arrival among non-U.S.-born persons.

Complete data were available for 55 330/85 958 cases. Varying the serial interval and geographic proximity used to derive clusters, we consistently estimated *R*_m_<1.0 and *k* < 0.08; the low value of *k* indicates a small number of source cases produce a disproportionate number of secondary cases.

U.S. TB reproductive number has a highly skewed distribution, indicating a minority of source cases disproportionately contribute to transmission.

## Introduction

Globally, 10 million people became sick with tuberculosis (TB) in 2019 [1]. In order to achieve the Sustainable Development Goal of ending the TB epidemic by 2030 [2], substantial reductions in TB incidence will be required. Understanding which control strategies are most effective at reducing TB incidence is of paramount importance to meet this goal.

Mathematical models are useful in determining the efficacy of prevention strategies at reducing disease incidence. However, the validity of these models relies on the values and distributions of input parameters, such as the effective reproductive number (*R*), the number of secondary cases of disease an individual source case produces. Mathematical models are often encoded with the assumptions that all individuals are equally infectious, which is questionable based on outbreak reports and molecular and spatial analyses [3–5]. The negative binomial dispersion parameter, *k*, quantifies the magnitude of overdispersion in the mean of *R* (*R*_m_). In the context of infectious disease transmission, smaller values of *k* (<0.10) indicate greater overdispersion in *R*_m_ and thus a higher degree of individual heterogeneity in transmission.

Molecular genotyping of *M. tuberculosis* isolates is often used to track TB transmission, decipher epidemiologic links between TB cases and derive TB transmission clusters [6]. In turn, these transmission clusters can be analysed to infer *k* [6–9]. Employing molecular genotyping and contact tracing data, several studies have observed substantial heterogeneity in observed secondary cases of TB [7–9]. We assess heterogeneity in infectiousness and estimate *R*_m_ and *k* using spoligotype and 24-locus mycobacterial interspersed repetitive units variable number tandem repeats (MIRU-VNTR) genotyping data from the U.S. Centers for Disease Control and Prevention (CDC). We hypothesise there is substantial heterogeneity in TB transmission in the United States.

## Methods

### Previous work estimating *R*_m_ and *k* for tuberculosis

We follow the approach of Ypma *et al*. [7]. In brief, they used insertion sequence (IS) *6110* restriction fragment length polymorphism (RFLP) genotyping data from the Netherlands to quantify heterogeneity in R. This approach requires deriving genotype clusters, defined as cases with matching molecular typing, then deriving transmission clusters with the following rules: cases with a unique genotype not observed in the 24 months before diagnosis comprise a new transmission cluster where the first temporal case in each cluster is the index case; cases with recent arrival (<6 months) to the country were considered index cases themselves, thus an individual transmission cluster could have >1 index case; and clusters occurring at the temporal beginning and end of the study were censored, effectively assuming these clusters are a size of ≥*y*, where *y* represents the cluster size, due to the potential for partially observed clusters. Using these rules to derive clusters, Ypma and colleagues then modelled TB transmission as a subcritical branching process whereby the number of secondary cases from an infectious TB case is modelled using a negative binomial offspring distribution with a mean R and dispersion parameter *k*. Maximum likelihood estimation is used to estimate *R*_m_ and *k*. Briefly, Ypma *et al*. constructed the likelihood model of observing cluster sizes given the number of index cases in all clusters as follows:
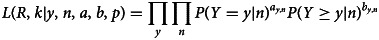


The likelihood function is a function of the parameters *R* and *k* (the parameter *p*, which represents the probability of observing non-index cases). The probability functions of *P*(*Y* = *y*│*n*) and *P*(*Y*⩾*y*│*n*) quantify the probabilities of observing a cluster size equal to *y* and at least *y* given n index cases in the cluster. Therefore, given the observed data *y* (cluster sizes), *n* (the numbers of index cases), *a* (*y*,*n*) (the number of clusters whose sizes are equal to *y* and numbers of index cases are equal to *n*), *b* (*y*,*n*) (the number of clusters whose sizes are at least y and numbers of index cases are equal to *n*), the maximum likelihood estimates of *R* and *k* can be obtained.

We modify this approach using molecular genotyping data from U.S. cases and incorporate geographic proximity of cases, disease site as a proxy for infectiousness and a more nuanced estimate of the serial interval, the duration of time between disease symptom onset of a case and its infector.

### Data source

We used spoligotype and 24-locus MIRU-VNTR data from the CDC's National Tuberculosis Genotyping Service (NTGS) and surveillance data from the National Tuberculosis Surveillance System (NTSS) for cases reported to CDC during 1 January 2009–31 December 2018. Cases from the 50 U.S. states and Washington D.C. were included if they had complete data on genotype and case characteristics, including disease site, month and year of U.S. arrival and reporting jurisdiction. Analysis of surveillance data was determined by CDC as non-human subjects research and did not require institutional review board approval.

### Derivation of genotype and transmission clusters

We defined cases as being in the same genotype cluster if the 24-loci MIRU-VNTR and spoligotype were an exact match. Cases with missing or mixed loci and cases that did not have genotyping data were excluded from the analysis.

We used Ypma *et al*.'s rules, summarised above, to split genotype clusters into transmission clusters [7]. Because cases across distanced locations might not be linked, we split genotype clusters by county Federal Information Processing Standard codes and by state in sensitivity analyses. We did not consider a case of extrapulmonary disease as the first case in any cluster because most extrapulmonary TB is noninfectious [10]. This rule resulted in clusters without a designated index case. Because the transmission model requires ≥1 index case per transmission cluster, we imputed these clusters to have one index case in our primary analysis. In sensitivity analyses, we excluded extrapulmonary TB cases.

Rather than assuming a 24-month serial interval, we implemented a probabilistic approach whereby the serial interval was selected randomly from a gamma distribution of median 0.51 years (95% credible interval: 0.41–0.64, shape = 1.47, rate = 0.1968). This distribution is based on previous work estimating the serial interval for TB in the U.S. [11]. We rounded serial interval estimates to the closest month to conform to month-level reporting in our data, resulting in a median time between cases of 6 months (95% simulation interval: 1–20). Using the serial interval distribution, we report the median and 95% simulation intervals across estimates.

## Results

### Case characteristics

There were 85 958 TB cases reported to NTSS from the 50 U.S. states and Washington D.C. during 1 January 2009–31 December 2018. We excluded 30 628 cases (36%), of which 72% (*N* = 22 121) lacked complete genotyping data (cases had mixed or missing loci or spoligotype, were culture-negative or an isolate was unavailable for genotyping).

Other exclusions included 19% (*N* = 5787) for mixed or missing genotype, 9% (*N* = 2657) for missing date of U.S. arrival and 0.2% (*N* = 63) for missing epidemiologic data. Extrapulmonary TB was more common among excluded cases than included cases (Supplemental Digital Content); other case characteristics were comparable. Of 55 330 remaining cases, 46 470 had pulmonary disease or both pulmonary and extrapulmonary disease and two-thirds of patients (66%) were non-U.S. born (Table 1).

**Table 1.** Characteristics of TB cases with complete MIRU-VNTR and spoligotype genotyping notified in the United States, 1 January 2009–31 December 2018 (*N* = 55 330)

Characteristic	*N* (%)
Disease site	
Pulmonary only	40 215 (72.68%)
Extrapulmonary only	8860 (16.01%)
Both	6255 (11.30%)
Age group (years)	
0–4	576 (1.04%)
5–14	530 (0.96%)
15–24	5839 (10.55%)
25–44	17 547 (31.71%)
45–64	17 297 (31.26%)
65+	13 541 (24.47%)
Origin of birth	
U.S. born	18 890 (34.14%)
Non-U.S. born^a^	36 440 (65.86%)
Time (years) since arrival to U.S. among non-U.S. born^b^	−5.9 (−18.9, 1.1)

a Includes persons born outside the United States (including U.S. territories), except persons born to at least one U.S. citizen parent.

b Median (25^th^ percentile, 75^th^ percentile).

### *R*_m_ and *k* estimates assuming a fixed 24-month serial interval

**Fig. 1.**
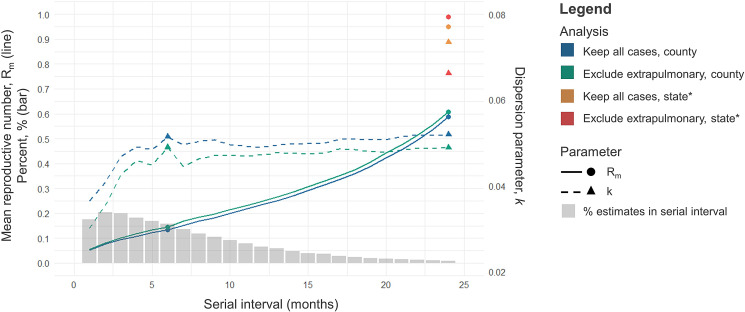
Estimation of mean reproductive number and dispersion parameter, *k*, among TB cases with 24-locus MIRU-VNTR and spoligotyping data in the United States, 2009–2018. Estimates for *R*_m_ (circles) and *k* (triangles) using the deterministic approach assuming a 24-month serial interval are indicated at the 24-month mark on the *x*-axis. Marker colours differentiate values for *R*_m_ and *k* using varying assumptions of the subset of cases included (keep all cases in blue and orange, exclude extrapulmonary TB cases in green and red) and the geographic unit used to split exact-match genotype clusters (county-level in blue and green, state-level in yellow and red). Estimates for *R*_m_ and *k* using a probabilistic approach whereby we applied a serial interval distribution (indicated in grey bars) with a mean serial interval of 6 months are indicated by solid (*R*_m_) and dotted (*k*) lines. The mean values of the probabilistic approach are indicated by a circle (*R*_m_) and triangle (*k*) at the 6-month mark on the *x*-axis. Probabilistic analyses used the county-level geographic unit (blue and green). *Not included in probabilistic analysis.

In the primary analysis assuming a fixed 24-month serial interval, keeping both pulmonary and extrapulmonary TB cases (*N* = 55 330 cases) and splitting genotype clusters at the county-level resulted in 45 386 transmission clusters with an estimated *R*_m_ = 0.59 and *k* = 0.05 (Fig. 1, 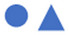). Splitting clusters by state resulted in 38 544 transmission clusters and an estimated *R*_m_ = 0.95 and *k* = 0.07 (Fig. 1, 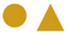). These results did not change substantially when we excluded extrapulmonary cases (*N* = 46 470 cases) and split genotype clusters by county (*N* = 37 909 transmission clusters, *R*_m_ = 0.61, *k* = 0.05) (Fig. 1, 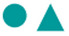) or state (*N* = 32 195 transmission clusters, *R*_m_ = 0.99, *k* *=* 0.07) (Fig. 1, 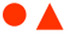).

### *R*_m_ and *k* estimates using a probabilistic approach for the serial interval

Using a probabilistic approach for the serial interval, keeping both pulmonary and extrapulmonary TB cases (*N* = 55 330 cases) and splitting genotype clusters at the county-level resulted in 49 215 (95% SI: 45 895–52 403) transmission clusters with an estimated median *R*_m_ = 0.13 (95% SI: 0.05–0.42) and *k* = 0.05 (0.04–0.05) (Fig. 1). Excluding extrapulmonary TB cases (*N* = 46 470 cases) resulted in 41 208 (95% SI: 38 354–46 470) transmission clusters and an estimated median *R*_m_ = 0.14 (95% SI: 0.06–0.44) and *k =* 0.04 (95% SI: 0.03–0.05) (Fig. 1).

## Discussion

We consistently estimated low values of *k*, indicating substantial heterogeneity and overdispersion in *R*_m_. These findings suggest a minority of source cases disproportionately contribute to TB transmission in the United States.

Our results affect TB transmission modelling research and public health practice. First, TB transmission models, often used to estimate *R* and inform prevention strategies, must be appropriately parameterised to maximise validity. A systematic review identified inconsistent TB serial interval and R estimates, which might be due to the diverse assumptions applied to parameters such as the transmission rate [12]. The implications of overdispersion in *k* for TB modelling is that TB transmission rate constants may more accurately summarise actual transmission when specified by a negative binomial distribution capturing individual transmission heterogeneity [13]. Second, our results point to the possibility that directing targeted prevention interventions towards the minority of source cases who disproportionately transmit TB might effectively reduce new infections. However, identifying these source cases is difficult, as transmission relies on complex host−pathogen interactions. While numerous case reports document super spreading events and host and pathogen characteristics associated with them [3, 4, 14, 15], few studies have attempted to identify characteristics giving rise to super spreading events in large cohorts [9].

Our conclusion is consistent with Ypma and colleagues' [7], despite differences between settings and study designs. We extended their work to incorporate the geographic proximity of cases to ensure the fidelity of transmission clusters. Deterministically splitting genotype clusters by state and county yielded slightly different estimates of *R*_m_ and *k,* reinforcing the importance of U.S. geography. However, estimates from both analyses indicated substantial overdispersion. Future work could incorporate a linear spatial scale to estimate the probability cases with matching genotypes are linked via transmission. Additionally, our results were similar to Ypma and colleagues despite using MIRU-VNTR and spoligotype. MIRU-VNTR and spoligotype have replaced RFLP as the gold standard for routine, large-scale genotyping studies because of the formers' quick turnaround and discriminatory power. Numerous studies have confirmed the methods are comparable [16–18], thus differences between estimates are likely not attributed to the form of molecular typing used. Both our study and Ypma and colleagues' were based in high-income, low burden TB settings, thus our conclusions are likely generalisable to similar settings. Conducting a similar analysis in a low-income, high burden TB setting is limited by the availability of genotyping data from such settings.

Our study limitations pertain to inherent constraints of making transmission inferences from molecular typing data. First, whole-genome sequencing (WGS) is newer technology with higher molecular resolution to better understand transmission [19, 20]. Because CDC began universal WGS of culture-positive *M.tb* isolates in 2018, WGS data over an extended time period are not yet available. While WGS provides some advantage over MIRU-VNTR, determining whether cases sharing the same genetically indistinguishably strain are in the same transmission cluster remains a challenge [21]. To improve our ability to elucidate these transmission clusters, we augmented genotyping data with epidemiologic data. Second, all molecular typing methods rely on culture-positive sputum specimens. We excluded approximately 25% of cases missing genotyping data, most of which were culture-negative. Recent advances to capture *M.tb* DNA directly from sputum specimens – without the need for culture – are in development and could improve our understanding of TB transmission. Similarly, we cannot rule out the likely occurrence of missed TB cases, despite robust public health efforts to diagnose TB and trace contacts of TB cases in the U.S. If missed cases were primarily in large clusters, this could underestimate the magnitude of overdispersion. Third, we estimate *R*_m_ based on matching molecular profiles across cases. While *R*_m_ is meant to represent the true underlying transmission cluster, it should not be confused to represent the effective reproduction number. Estimation of *k* was consistent across models, however, we found *R*_m_ to be sensitive to model inputs, *R*_m_ is thus not a reliable indicator of the number of secondary cases from an individual with TB and should not be interpreted as such. Lastly, we select the first temporal case in a cluster as the index case, which is likely an oversimplification of the underlying true order of cases in a transmission chain. However, this assumption is not a source of bias because the method we applied estimates *k* based on the cluster size and number of index cases, not necessarily who is selected as an index case in a given cluster. Inferences based on characteristics of index cases – which we do not report – should not be made.

We found substantial individual variation in the number of secondary TB cases from a source case. Improving the validity of TB transmission models requires incorporating heterogeneity when specifying model parameters. Prevention strategies accounting for highly heterogeneous transmission processes might be most effective for reducing TB incidence. Increased understanding of host and pathogen factors affecting transmission probability will be required to develop and implement optimal prevention strategies.

## Data Availability

The data contain information abstracted from the national TB case report form called the Report of Verified Case of Tuberculosis (RVCT) (OMB No. 0920-0026). These data have been reported voluntarily to CDC by state and local health departments, and are protected under the Assurance of Confidentiality (Sections 306 and 308(d) of the Public Health Service Act, 42 U.S.C. 242k and 242 m(d)), which prevents disclosure of any information that could be used to directly or indirectly identify patients. For more information, see the CDC/ATSDR Policy on Releasing and Sharing Data (at http://www.cdc.gov/maso/Policy/ReleasingData.pdf). A limited dataset is available at http://wonder.cdc.gov/TB-v2013.html. Researchers seeking additional data may apply to analyse National TB Surveillance System data at CDC headquarters by contacting TBInfo@cdc.gov.
